# Transcranial magnetic stimulation neurophysiology of patients with major depressive disorder: a systematic review and meta-analysis

**DOI:** 10.1017/S0033291720004729

**Published:** 2021-01

**Authors:** Megumi Kinjo, Masataka Wada, Shinichiro Nakajima, Sakiko Tsugawa, Tomomi Nakahara, Daniel M. Blumberger, Masaru Mimura, Yoshihiro Noda

**Affiliations:** 1 Department of Neuropsychiatry, Keio University School of Medicine, Tokyo, Japan; 2 Department of Psychiatry, Temerty Centre for Therapeutic Brain Intervention, Centre for Addiction and Mental Health, University of Toronto, Toronto, Canada

**Keywords:** cortical excitability, cortical inhibition, excitatory and inhibitory imbalance hypothesis, major depressive disorder, neuroplasticity hypothesis, TMS neurophysiology

## Abstract

Major depressive disorder (MDD) is a mental illness with high socio-economic burden, but its pathophysiology has not been fully elucidated. Recently, the cortical excitatory and inhibitory imbalance hypothesis and neuroplasticity hypothesis have been proposed for MDD. Although several studies have examined the neurophysiological profiles in MDD using transcranial magnetic stimulation (TMS), a meta-analysis of TMS neurophysiology has not been performed. The objective of this study was to compare TMS-electromyogram (TMS-EMG) findings between patients with MDD and healthy controls (HCs). To this end, we examined whether patients with MDD have lower short-interval cortical inhibition (SICI) which reflects gamma-aminobutyric acid (GABA)_A_ receptor-mediated activity, lower cortical silent period (CSP) which represents GABA_B_ receptor-mediated activity, higher intracortical facilitation (ICF) which reflects glutamate N-methyl-D-aspartate receptor-mediated activity, and the lower result of paired associative stimulation (PAS) paradigm which shows the level of neuroplasticity in comparison with HC. Further, we explored the effect of clinical and demographic factors that may influence TMS neurophysiological indices. We first searched and identified research articles that conducted single- or paired-pulse TMS-EMG on patients with MDD and HC. Subsequently, we extracted the data from the included studies and meta-analyzed the data with the comprehensive meta-analysis software. Patients with MDD were associated with lower SICI, lower CSP, potentially higher ICF, and lower PAS compared with HC. Our results confirmed the proposed hypotheses, suggesting the usefulness of TMS neurophysiology as potential diagnostic markers of MDD.

## Introduction

### Overview of depression

Major depressive disorder (MDD) is one of the most common psychiatric disorders, which affects >264 million people worldwide (GBD, 2017 Disease & Injury Incidence…, 2018). Depression is associated with a high mortality rate, with a hazard ratio of 1.61 (Pratt, Druss, Manderscheid, & Walker, 2016) and a particularly high suicide rate. These factors, in part, have contributed to the large societal, medical and economic burden of this disease. The first line of treatment for MDD includes psychotherapy and pharmacotherapy; however, at least one-third of patients are resistant to these treatments (Ionescu, Rosenbaum, & Alpert, 2015). Therefore, it is important to elucidate the pathophysiology of MDD to develop effective strategies for treatment-resistant depression.

### Disrupted excitatory and inhibitory balance in MDD

Several lines of evidence suggest that there is an imbalance between cortical excitability and inhibition in patients with MDD, whereby there is excessive cortical excitability and reduced cortical inhibition (Gabbay et al., 2017; Sanacora, Treccani, & Popoli, 2012). For example, previous studies have reported decreased gamma-aminobutyric acid (GABA) concentrations and increased glutamate concentrations in the brain of patients with MDD, using proton magnetic resonance spectroscopy (^1^H-MRS) (Bhagwagar et al., 2008; Moriguchi et al., 2019; Sanacora et al., 2004). In addition, ketamine, which is a glutamate N-methyl-D-aspartate (NMDA) receptor antagonist, represents an effective treatment approach for treatment-resistant depression (McGirr et al., 2015). Taken together, these findings suggest that patients with depression may have disrupted GABA and glutamate NMDA receptor-mediated activity. Furthermore, functional magnetic resonance imaging studies have noted a reduction of functional connectivity in several brain regions including the prefrontal cortex (PFC) (Zeng et al., 2012), as well as a reduction of PFC volumes in patients with MDD (Botteron, Raichle, Drevets, Heath, & Todd, 2002). These findings support the notion that neuroplasticity in the PFC may be lower in patients with MDD compared to healthy controls (HCs) (Noda et al., 2018; Pittenger & Duman, 2008).

### Transcranial magnetic stimulation (TMS) neurophysiological paradigms

Cortical excitability, inhibition, and neuroplasticity can be measured by TMS paradigms. Output measures of TMS can be assessed in two ways: coupling of TMS with peripheral electromyography (EMG) or with concurrent electroencephalography (EEG) (Farzan et al., 2013). Single- and paired-pulse TMS paradigms have been shown to assess intracortical facilitation (ICF), and intracortical inhibition, which includes short-interval cortical inhibition (SICI) and long-interval cortical inhibition (LICI) (Chen, 2000). SICI consists of a subthreshold condition pulse and suprathreshold test pulse with an interstimulus interval of 1–5 ms and is thought to reflect GABA_A_ receptor-mediated activity (Hanajima et al., 1998; Ilić et al., 2002; Ziemann, Rothwell, & Ridding, 1996). LICI is composed of a suprathreshold condition pulse and test pulse with an interstimulus interval of 100–200 ms (Nakamura, Kitagawa, Kawaguchi, & Tsuji, 1997). LICI is thought to reflect GABA_B_ receptor-mediated activity. GABA_B_ activity can also be measured using a TMS paradigm known as cortical silent period (CSP), whereby a strong test pulse is delivered during a voluntary muscle contraction (McDonnell, Orekhov, & Ziemann, 2006; Siebner, Dressnandt, Auer, & Conrad, 1998; Wilson, Lockwood, Thickbroom, & Mastaglia, 1993). In contrast to these measures, ICF is thought to be a measure of cortical excitability, specifically glutamate NMDA receptor-mediated activity (Hunt & Castillo, 2012). ICF consists of a subthreshold condition pulse and suprathreshold test pulse with an interstimulus interval of 10–15 ms (Liepert, Schwenkreis, Tegenthoff, & Malin, 1997; Ziemann et al., 1996). An additional TMS paradigm called short-latency afferent inhibition (SAI) is an index of the central cholinergic activity (Tokimura et al., 2000). SAI is measured by delivering TMS over the M1 immediately after contralateral peripheral median nerve stimulation, which attenuates the motor-evoked potential (MEP) (Tokimura et al., 2000). Furthermore, neuroplasticity can be indexed using a TMS paradigm called paired associative stimulation (PAS). This paradigm combines repeated electrical stimulation to the peripheral median nerve of the wrist with TMS to the contralateral primary motor cortex, for over 30 min (Stefan, Kunesch, Cohen, Benecke, & Classen, 2000). Depending on the time interval between the PNS and TMS pulses, PAS can induce either long-term potentiation (LTP)-like (e.g. ~25 ms interval) and long-term depression (LTD)-like (e.g. ~10 ms interval) neuronal activity (Buonomano & Merzenich, 1998).

### Previous studies of TMS neurophysiological paradigms in MDD

As mentioned earlier, research suggests that patients with MDD may have excessive cortical excitability and reduced cortical inhibition, in addition to lower levels of neuroplasticity in the PFC. Several neurophysiological studies using TMS in patients with MDD have attempted to establish support for these hypotheses (Kaskie & Ferrarelli, 2018); however, the results of these studies are inconsistent. Therefore, continued research is necessary in order to elucidate if these hypotheses are valid.

### Aim of this review study

Here, we conducted a systematic review and meta-analysis to compare TMS-EMG indices between patients with MDD and HC. Our hypotheses were as follows: patients with MDD would have lower GABA_A/B_ receptor-mediated activity, higher glutamate NMDA receptor-mediated activity, and lower levels of neuroplasticity compared to HC. In addition, we explored the effects of clinic-demographic factors such as age, sex, and depression severity on the TMS findings in patients with MDD.

## Methods

### Search strategy

Research articles written in English were screened by three reviewers using EMBASE, Medline, and PsycINFO from the earliest record to 29 April 2019. The search terms included ‘non-invasive brain stimulation’ or ‘TMS’ or ‘transcranial magnetic stimulation’, ‘brain activity’ or ‘brain waves’ or ‘EEG’ or ‘electroencephalogram’ or ‘electroencephalography’ or ‘EMG’ or ‘MEP’ or ‘motor evoked potential’ or ‘neurophysiolo’ or ‘neuroplasticity’ or ‘plasticity’ or ‘plastic’ or ‘short interval intracortical inhibition’ or ‘SICI’ or ‘intracortical facilitation’ or ‘ICF’ or ‘long interval intracortical inhibition’ or ‘LICI’ or ‘paired associative stimulation’ or ‘PAS’ or ‘short latency afferent inhibition’ or ‘SAI’ or ‘contralateral silent period’ or ‘CSP’, and ‘depression’.

### Inclusion criteria

Studies were included in the analysis if they met the following criteria: (1) depression was diagnosed by operational diagnostic criteria; (2) TMS-EMG was conducted using any of the following paradigms, SICI, LICI, ICF, SAI, PAS, or CSP; and (3) results were included for both patients with depression and HCs. Various types of depression, such as atypical depression and melancholic depression, were also included. Vascular depression (VD) was excluded from the main meta-analyses, however, we included VD to sub-analyze its effect on the results for certain TMS paradigms. Of note, any discrepancies on the data extraction process were reviewed and resolved by the senior researcher (Y.N.).

### Analysis

The meta-analyses and meta-regression analyses were conducted using the comprehensive meta-analysis (CMA) Software (Biostat Inc.). Outcome variables were denoted as standardized mean differences (SMD). A 95% confidence interval (CI) was calculated following summary statistics. Study heterogeneity was evaluated using the *I^2^* statistic with *I*^2^ ≥ 50% indicating significant heterogeneity. When a two-sided *p* value was <0.05, it was statistically considered to be significant. Further, we conducted meta-regression analyses to examine the effects of additional factors including patients' age, gender rate, and severity of depression. For the severity of the depression factor, the Hamilton Rating Scale for Depression (HRSD) with 17 items was selected for the moderator variable. Studies that included the Montgomery–Åsberg Depression Rating Scale (MADRS), Beck Depression Inventory (BDI), or BDI-II scores, were converted to HRSD scores, as there are strong correlations between HRSD score and correlation coefficients of *r* = 0.88, *r* = 0.73, and *r* = 0.74, respectively (Furukawa et al., 2019; Heo, Murphy, & Meyers, 2007). The formula used for the conversion of the MADRS to the HRSD was: HDRS17 = −1.58 + 0.86 × MADRS (Heo et al., 2007). For the conversion of scores on the BDI or BDI-II to the HRSD, published data from a previous study were used (Furukawa et al., 2019, Table 2).

For the included studies with missing data values, we supplemented them using one of the following options: (1) contacting the authors for additional data or (2) enlarging the graphic charts, if present, and measuring the data values with R Studio Software or a ruler. Thus, we also conducted the analyses only on the studies that had complete data and confirmed if the pattern of findings held the same.

### Risk of bias of the included studies

We used the risk of a bias assessment tool for non-randomized studies (Kim et al., 2013) to assess the risk of bias for the following factors: the selection of participants, confounding variables, measurement of exposure, blinding of outcome assessments, incomplete outcome data, and selective outcome reporting. The assessment is shown in online Supplementary Fig. S1.

### Publication bias

The publication bias was assessed by Egger's test using the CMA Software.

## Results

Out of 882 initial records, 16 studies were included in this meta-analysis. The Preferred Reporting Items for Systematic Reviews and Meta-analyses Statement flow diagram is presented in online Supplementary Fig. S2. The characteristics of the included studies are detailed in Table 1. There were nine studies which measured SICI (Bajbouj et al., 2006; Concerto et al., 2013; Croarkin et al., 2013; Lefaucheur et al., 2008; Levinson et al., 2010; Lewis et al., 2018; Maeda, Keenan, & Pascual-Leone, 2000a, 2020b; Münchau et al., 2005; Veronezi et al., 2016), two studies for LICI (Croarkin et al., 2014; Lewis et al., 2018), nine studies for CSP (Bajbouj et al., 2006; Concerto et al., 2013; Croarkin et al., 2013; Lefaucheur et al., 2008; Levinson et al., 2010; Lewis et al., 2018; Münchau et al., 2005; Steele, Glabus, Shajahan, & Ebmeier, 2000; Veronezi et al., 2016), nine studies for ICF (Bajbouj et al., 2006; Concerto et al., 2013; Croarkin et al., 2013; Lefaucheur et al., 2008; Levinson et al., 2010; Lewis et al., 2018; Maeda et al., 2000a, 2020b; Münchau et al., 2005; Veronezi et al., 2016), and three studies for PAS (Bhandari et al., 2018; Kuhn et al., 2016; Player et al., 2013). There were no studies that examined the SAI paradigm that met the inclusion criteria. Due to the insufficient number of studies, we did not conduct the meta-analysis on the LICI and SAI paradigms.

**Table 1.** Characteristics of included studies

Author Year	*N* (Controls/patients)	Age (SD) (controls/patients)	%female (controls/patients)	Depression severity (SD)	Patients' characteristics (medicated or unmedicated, VD, etc)	Key findings
Bajbouj et al. (2006)	20/20	44 (14.6)/42.9 (11.9)	30/30	HRSD-24: 21.1 (6)	Unmedicated	Lower SICI in patient group, shorter CSP in patient group, no difference in ICF between groups
Bella et al. (2011)	10/15	67.7 (3.9)/70.5 (6.6)	50.0/53.3	HRSD-17: 14.9 (6.4)	VD	No difference in SICI, CSP, and ICF between groups
Bhandari et al. (2018)	34/48	69.1 (8.9)/67.9 (6.9)	58.8/60.4	MADRS: 25.9 (4.9)	Late life depression	Lower neuroplasticity induced by PAS in patient group
Concerto et al. (2013)	11/11	67.36 (3.75)/62.45 (7.64)	45.5/50.0	HRSD-17: MDD 20.27 (4.41), VD 15.91 (7.23)	Divided into MDD group and VD group	No difference in SICI and ICF between patient group and control group, longer CSP in MDD group and no difference in CSP between VD group and control group
Croarkin et al. (2013)	22/24	13.77 (2.18)/13.87 (2.11)	50.0/58.3	CDRS-R: 58.9 (8.5)	Unmedicated	No difference in SICI and CSP between groups, higher ICF in patient group
Croarkin et al. (2014)	19/14	13.9 (2.2)/14.0 (2.1)	57.9/57.1	CDRS-R: 59.0 (9.6)	Unmedicated	No difference in LICI between groups
Kuhn et al. (2016)	27/27	37.3 (10.3)/38.7 (11.2)	48.1/44.4	BDI-II: 23.6 (NA)	Medicated	No difference in neuroplasticity induced by PAS between groups
Lefaucheur et al. (2008)	35/35	43 (NA)/56 (16.6)	51.4/60.0	MADRS: 32.1 (8.3)	Medicated	Lower SICI in patient group, no difference in CSP and ICF between groups
Levinson et al. (2010)	25/60	43.84 (8.95)/47.17 (11.2)	52.0/63.3	HRSD-17: 18.6 (11.4)	Divided into TRD group, unmedicated group and medicated euthymic group	No difference in SICI and ICF between groups, shorter CSP in patient group
Lewis et al. (2018)	20/54	14.2 (1.76)/15.8 (1.78)	45.0/64.8	CDRS-R: 42.9 (13.9)	Divided into depression groups with and without suicidal behavior	No difference in SICI, LICI, and ICF between groups, shorter CSP in patient group
Maeda et al. (2000a, 2000b)	8/8	44.9 (NA)/46.8 (NA)	25.0/37.5	BDI: 21.5 (11.6)	TRD and unmedicated	No difference in SICI and ICF between groups
Münchau et al. (2005)	6/7	39.0 (NA)/43.6 (NA)	33.0/71.4	BDI: 56.8 (5.9)	Depressed epilepsy patients and medicated	No difference in SICI and CSP between groups, higher ICF in patient group
Pennisi et al. (2016)	15/16	63.8 (7.2)/68.1 (8.6)	NA	HRSD-17: 14.8 (6.1)	VD	Higher SICI in patient group, no difference in CSP and ICF between groups
Player et al. (2013)	23/23	38.5 (13.1)/38.0 (12.8)	56.5/56.5	MADRS: 30.7 (3.4)	NA	Lower neuroplasticity induced by PAS in patient group
Steele et al. (2000)	19/16	40.17 (11.5)/43.1 (8.9)	68.4/75.0	BDI: 24.9 (8.8)	NA	longer CSP in patient group
Veronezi et al. (2016)	21/60	28.0 (8.0)/37.7 (9.3)	47.6/68.3	HRSD-17: 21.7 (3.8)	Divided into atypical, melancholic, and undifferentiated MDD groups	no difference in SICI and ICF between groups, shorter CSP in patient group

MDD, major depressive disorder; VD, vascular depression; TRD, treatment-resistant major depressive disorder; HRSD, Hamilton Rating Scale for Depression; MADRS, Montgomery–Åsberg Depression Rating Scale; CDRS-R, Children's Depression Rating Scale-Revised; BDI, Beck Depression Inventory; BDI-II, Beck Depression Inventory second edition; SICI, short-interval cortical inhibition; LICI, long-interval cortical inhibition; CSP, cortical silent period; ICF, intracortical facilitation; PAS, paired associative stimulation.

### Meta-analysis

**Fig. 1.**
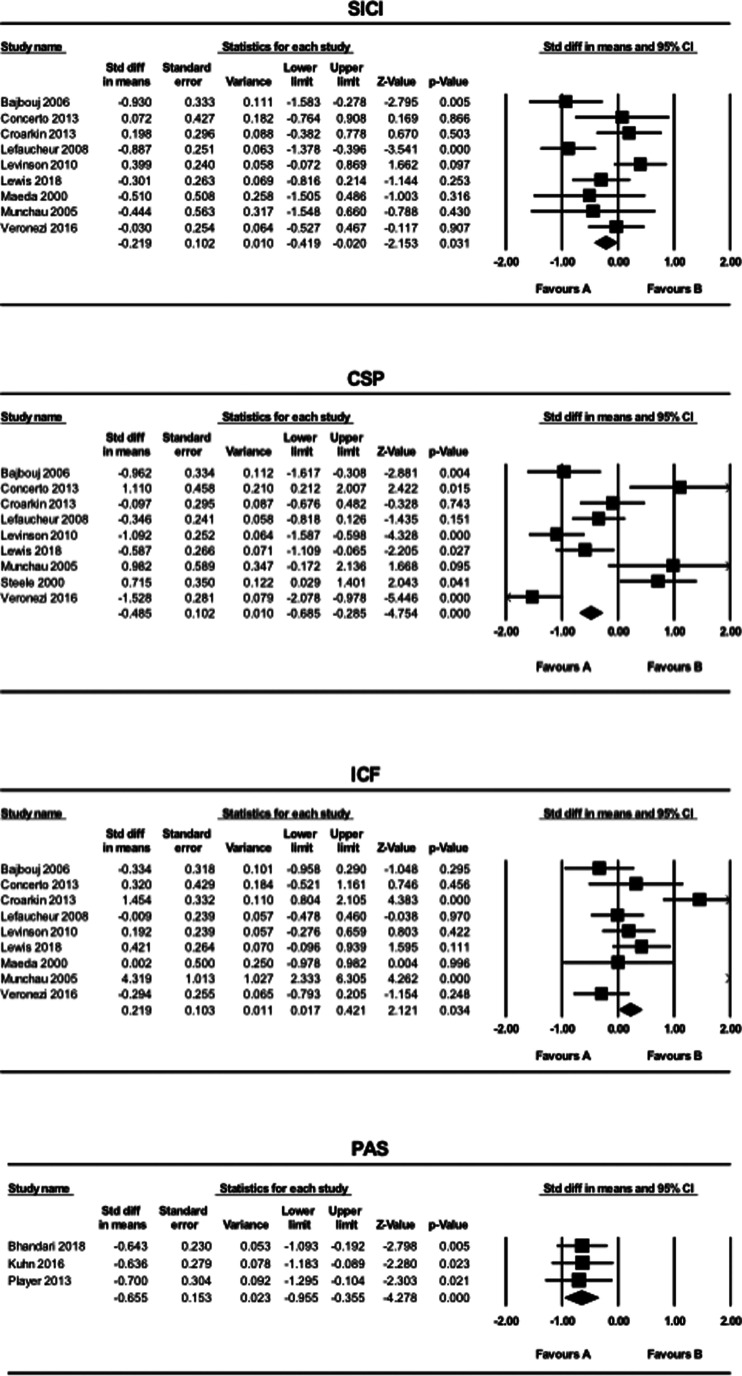
The results of meta-analyses for the SICI, LICI, CSP, ICF, and PAS paradigms comparing patients with MDD and HCs. Favors A (left side): HC. Favors B (right side): MDD.

The results of the meta-analysis for SICI, CSP, ICF and PAS are shown in Fig. 1.

SICI and CSP values were smaller in the MDD group compared to the HC group (SICI: SMD = −0.22, CI −0.42 to −0.020, *p* = 0.031; CSP: SMD = −0.49, CI −0.69 to −0.29, *p* < 0.001). In contrast, ICF values were greater in patients with MDD compared to HCs (SMD = 0.22, CI 0.017–0.42, *p* = 0.034). MEP values generated using the PAS paradigm were smaller in the MDD group compared to the HC group (SMD = −0.66, CI −0.96 to −0.36, *p* < 0.001). Further, for the PAS paradigm, all of the three studies included in this systematic review indicated LTP-like activity.

### The analyses when the studies with missing data values were excluded

There were four studies measuring SICI (Concerto et al., 2013; Levinson et al., 2010; Maeda et al., 2000a, 2020b; Münchau et al., 2005), one study for CSP (Levinson et al., 2010), three studies for ICF (Levinson et al., 2010; Maeda et al., 2000a, 2020b; Münchau et al., 2005), and two studies for PAS (Bhandari et al., 2018; Player et al., 2013) which had missing data values. Thus, we measured the values from graphic charts in the articles using R Studio Software or a ruler. When these studies with missing data values were excluded from the analyses, the pattern of the findings still remained. However, the finding of ICF became non-significant in this analysis (SICI: SMD = −0.38, CI −0.62 to −0.14, *p* = 0.0020; CSP: SMD = −0.37, CI −0.59 to −0.15, *p* = 0.0010; ICF: SMD = 0.18, CI −0.050 to 0.41, *p* = 0.12). On the other hand, we could not conduct the analysis of the PAS paradigm since there was only one study left.

### The effect of VD on the results

**Fig. 2.**
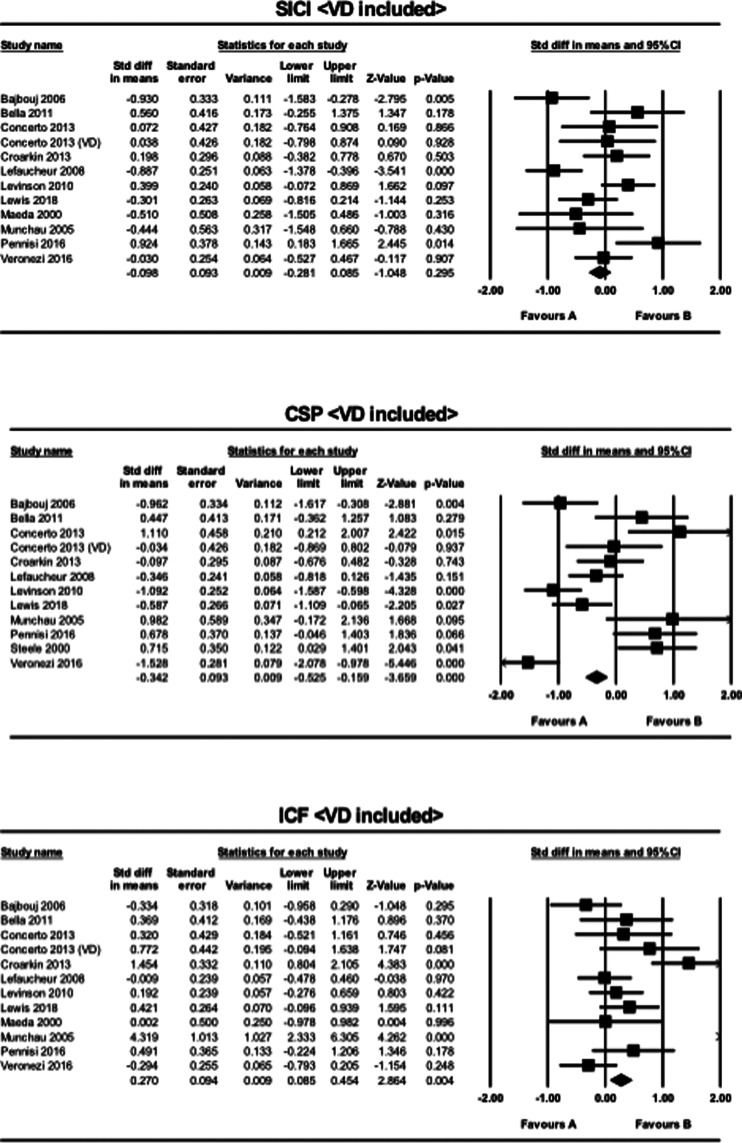
The results of meta-analyses for the SICI, CSP, and ICF paradigms, when VD was included in MDD. Favors A (left side): HC. Favors B (right side): MDD.

Three studies employed the SICI, CSP, and ICF paradigms in patients with VD, while no studies were found in this population using the PAS paradigm. When VD was included for the analysis of the SICI paradigm, the result for the meta-analysis became non-significant (SMD = −0.098, CI −0.28 to 0.085, *p* = 0.30). For the analysis of the CSP paradigm, the result remained significant (SMD = −0.34, CI −0.53 to −0.16, *p* < 0.001). Similarly, for the ICF paradigm, the result remained significant when VD was included (SMD = 0.27, CI 0.085 to 0.45, *p* = 0.004) (Fig. 2).

### Meta-regression analysis

Meta-regression analyses were conducted for the SICI, CSP, and ICF paradigms, while they could not be conducted for the PAS paradigm due to the insufficient number of studies.

First, patients' age was not associated with the SMD of the SICI, CSP, or ICF paradigms between patients with MDD and HCs (SICI: slope = −0.0084, CI −0.032 to 0.015, *p* = 0.24; CSP: slope = 0.013, CI −0.027 to 0.052, *p* = 0.26; ICF: slope = −0.018, CI −0.049 to 0.012, *p* = 0.12). The scatter plots are displayed in online Supplementary Fig. S3.

Second, the proportion of female patients was not associated with the SMD of the SICI, CSP, or ICF paradigms between patients with MDD and HCs (SICI: slope = 0.019, CI −0.0076 to 0.045, *p* = 0.083; CSP: slope = 0.018, CI −0.028 to 0.063, *p* = 0.23; ICF: slope = 0.027, CI −0.012 to 0.066, *p* = 0.085). The scatter plots are displayed in online Supplementary Fig. S4.

Finally, the severity of depression as assessed by the HRSD-17 was not associated with the SMD of the SICI and CSP paradigms between patients with MDD and HCs (SICI: slope = −0.037, CI −0.10 to 0.030, *p* = 0.14; CSP: slope = 0.052, CI −0.074 to 0.18, *p* = 0.21), while it was associated with higher SMDs in the ICF paradigm (slope = 0.15, CI 0.048–0.26, *p* = 0.0023). The scatter plots are displayed in online Supplementary Fig. S5.

### Publication bias

Egger's test showed no publication bias in the analysis of the SICI, CSP, ICF, and PAS paradigms. The funnel plots are displayed in online Supplementary Fig. S6.

### Risk of bias

The risk of bias of the included studies is summarized in online Supplementary Fig. S1. For ‘random sequence generation’, the risk of bias was ‘unclear’ for four studies which did not mention the method of recruitment of participants. The risk of bias for ‘incomplete outcome data’ was ‘unclear’ for one study which did not specify how data was excluded. For ‘selective reporting’, the risk of bias was ‘unclear’ for all studies since we did not have access to the experimental protocols.

In addition, no specific sponsorship bias was identified for the included studies in this review, as none of the studies were funded by the private sector. Although some studies received funding from companies, those companies was not likely to affect the results because their business was not related to TMS neurophysiology. Other than these studies, some of the included studies mentioned that they were funded by some foundations which were not likely to make sponsorship bias. The other studies stated that they had no conflict of interest, or did not mention about conflict of interest.

## Discussion

This meta-analysis compared GABA_A/B_ receptor-mediated activity, glutamate NMDA receptor-mediated activity, and neuroplasticity between patients with MDD and HC through comprehensive TMS-EMG neurophysiological indices. Our analyses revealed that compared to HCs, patients with MDD have lower SICI, CSP, and probably higher ICF, with small effect sizes, and lower PAS, with a medium effect size (Fig. 1). These results suggest that patients with MDD have lower GABA_A/B_ receptor-mediated activity, a lower level of neuroplasticity, and might have higher glutamate NMDA receptor-mediated activity compared with HC. These findings provide support for the proposed cortical excitatory and inhibitory imbalance hypothesis and neuroplasticity hypothesis of MDD. Taken together, our results suggest that out of the five TMS paradigms, lower values of SICI, CSP, and PAS paradigms, and higher ICF could represent biomarkers for MDD, which can be used to distinguish MDD patients from HCs. In contrast to our ICF finding, a previous meta-analysis of glutamatergic neurometabolite levels in MDD as measured by ^1^H-MRS revealed decreased glutamate + glutamine levels in the medial frontal cortex in patients with MDD (Moriguchi et al., 2019). This discrepancy may be due to differences in the region of interest and the modality of measurement. For example, TMS measures functional neural dynamics, while ^1^H-MRS measures static neurometabolite levels. Thus, the combination of these two may represent a more comprehensive measure of the difference in glutamate NMDA receptor-mediated activity between patients with MDD and HC.

One TMS-EEG study examined neuroplasticity differences in PFC activity between patients with MDD and HC using the PAS paradigm (Noda et al., 2018). The study showed that prefrontal neuroplasticity was lower in patients with MDD compared to HCs (SMD = −0.78, CI −1.1 to −0.51, *p* = 0.004), supporting the result of the current meta-analysis findings for PAS in the motor cortex. These results indicate that reduced neuroplasticity is not limited to the motor cortex and might extend to broad cortical regions.

When studies of VD were included in the sub-analysis, the group differences in SICI no longer remained significant and differences in CSP became smaller. In contrast, the inclusion of the VD studies increased the group differences of the ICF paradigm (Fig. 2). The majority of patients with VD exhibit dementia-like pathology due to widespread microvascular insults, resulting in impaired inhibitory function. This is thought to lead to further disruption in cortical excitability and decrease in cortical inhibition (Alexopoulos et al., 1997; Issac, Chandra, & Nagaraju, 2013). Our meta-analyses indicated that patients with VD showed higher cortical excitability and higher cortical inhibition compared to the analysis where they were not included. Thus, our ICF findings were in line with previous research, whereas our analyses of SICI and CSP were not. This discrepancy is possibly due to the compensatory mechanism of interhemispheric inhibition. That is, when the excitability of one hemisphere increases (i.e. increased ICF), the inhibitory properties of the contralateral hemisphere also increases in response (i.e. increased SICI and CSP), and vice versa.

The results of meta-regression on patients' age suggest that cortical functions of the M1, including GABA_A/B_ and glutamate NMDA receptor-mediated activity, may not be significantly influenced by age (online Supplementary Fig. S3). In general, however, neurophysiological activities have been shown to decrease with age (Talelli, Ewas, Waddingham, Rothwell, & Ward, 2008). This discrepancy was possibly due to the small number of included studies. Further research with a larger number of studies is needed to confirm the effect of age on these neurophysiological indices.

The results of meta-regression on patients' sex suggest that cortical functions of the M1, including GABA_A/B_ and glutamate NMDA receptor-mediated activity, do not differ between males and females (online Supplementary Fig. 4). A previous TMS study found a significant difference in TMS-induced MEPs of the lower limbs but not of the upper limbs between males and females (Cantone et al., 2019). This may be because the sex difference in the distance of the corticospinal tract from the M1 to the upper limbs is relatively smaller compared to the lower limbs. In the present systematic review, all of the studies included in this meta-analysis assessed TMS-EMG of the upper limbs. Thus, MEPs measured from the upper limbs may not detect subtle sex differences. In contrast, a previous study explored the effects of female hormones such as estrogen and progesterone on cortical excitability and found significantly higher motor threshold values at the first dorsal interosseous muscle using TMS applied to the M1 in women with amenorrhea compared to women in the early follicular stage (Chagas et al., 2018). This, therefore, highlights a potential difference in cortical functions of the M1 between males and females. Our analysis did not include information regarding the menstrual cycle of the study samples due to the lack of information provided.

The result of meta-regression on the severity of depression for the ICF paradigm suggests that the worse the HRSD-17 score, the higher the glutamate NMDA receptor-mediated activity (online Supplementary Fig. S5). Therefore, ICF may represent a state marker of MDD. In contrast, the results of meta-regression on depression severity for the SICI and CSP paradigms show no correlations between HRSD-17 scores and GABA_A/B_ receptor-mediated activity (online Supplementary Fig. S5). Taken together, our results suggest that while SICI and CSP may be biomarkers of MDD, it is difficult to evaluate the state of depression from the inhibitory function of the corticospinal tract.

There are several novel therapeutic strategies for MDD that target the neurophysiological bases of MDD. For instance, ketamine, a non-competitive antagonist of the NMDA receptor (Anis, Berry, Burton, & Lodge, 1983), is now used in refractory depression at some specialized medical institutions. Since patients with MDD have higher ICF compared to HCs, ketamine may rapidly suppress hyperexcitation in glutamate NMDA receptor-mediated activity, resulting in improvement of depression symptoms.

Another promising pharmacological treatment for MDD is a novel GABA_A_ receptor positive allosteric modulator known as SAGE-217 (3*α*-hydroxy-3*β*-methyl-21-(4-cyano-1*H*-pyrazol-1′-yl)-19-nor-5*β*-pregnan-20-one) (Martinez Botella et al., 2017). Our SICI findings showing that GABA_A_ receptor-mediated activity may be lower in patients with MDD compared to HCs is suggestive of the effectiveness of SAGE-217 for the treatment of MDD.

Some neurosteroids have also been shown to affect the state of depression. For example, estrogen attenuates GABA_A/B_ receptor-mediated activities (Lagrange, Wagner, Rønnekleiv, & Kelly, 1996; Mukherjee et al., 2017). However, no relationship was found between the proportion of females in the included studies and GABA_A/B_ receptor-mediated inhibitory functions in the present meta-regression analysis (see online Supplementary Fig. S4). Another neurosteroid example is allopregnanolone, which is a positive allosteric modulator of the GABA_A_ receptor (Faroni & Magnaghi, 2011). Allopregnanolone also stimulates GABA synthesis by increasing the level of glutamic acid decarboxylase of 67 kDa, resulting in the activation of both GABA_A/B_ receptor-mediated activities (Magnaghi et al., 2010). The results of our analyses on SICI and CSP suggest the effectiveness of allopregnanolone as a potential treatment for MDD in women. In support of this, allopregnanolone has recently been approved by the FDA to treat postpartum depression, a condition that is associated with disrupted GABAergic functioning due to a rapid postpartum drop in progesterone (Walton & Maguire, 2019).

Other than pharmacotherapy, repetitive TMS (rTMS) has emerged as a promising treatment for treatment-resistant depression. rTMS is thought to exert its therapeutic effect through the induction of neuroplasticity in both excitatory and inhibitory synapses (Lenz et al., 2016). Our PAS analysis indicates a lower level of neuroplasticity in patients with MDD compared to HCs, thus suggesting that rTMS could be a useful treatment to target the underlying pathophysiological impairments associated with depression.

The present study has several limitations. First, we failed to perform the meta-analysis for the PAS due to the limited number of the included studies, warranting further research on PAS in MDD. Ongoing investigation as the field continues to grow would improve the accuracy and reliability of the results. Second, for some studies, we had to impute the results from figures using R Studio software or a ruler which also impacted the accuracy of the findings. When the studies with missing data were excluded from the analyses, the difference of the ICF results between HCs and patients with MDD became non-significant. Therefore, ICF findings in patients with MDD should be interpreted with caution at this time. Further research on the ICF paradigm is needed comparing larger sizes of patients with MDD and HCs. Third, the concomitant medication administered to patients with MDD was not standardized across the studies. Furthermore, as mentioned earlier, the effect of hormonal fluctuation due to the menstrual cycle in females on neurophysiological findings was not considered as a confounding factor. Additionally, there are other potential confounding factors that could affect the results, including, alcohol, drugs, smoking, and physical activity that were not measured in the studies (Huang et al., 2017; Kähkönen, Wilenius, Nikulin, Ollikainen, & Ilmoniemi, 2003; Kalivas & O'Brien, 2008). Finally, three studies using the scales of depression other than HRSD-17, MADRS, and BDI (Bajbouj et al., 2006; Croarkin et al., 2013; Lewis et al., 2018) could not be included in the meta-regression since the scales other than these three did not correlate with HRSD-17 score, which we selected as the moderator variable.

In summary, our results provided support for the cortical excitatory and inhibitory imbalance hypothesis as well as the neuroplasticity hypothesis. The present systematic review and meta-analyses on TMS neurophysiology in MDD warrants further research with larger sample sizes to replicate our findings and the consideration of potential confounding factors that may affect neural activity as mentioned above. Finally, given the results of this study, TMS neurophysiology has the potential not only to distinguish MDD from HCs but also to be a useful neuroscientific tool to elucidate the pathophysiology of MDD.

## Supplementary material

For supplementary material accompanying this paper visit https://doi.org/10.1017/S0033291720004729. click here to view supplementary material
