# Supplementary material for: Transcranial magnetic stimulation neurophysiology of patients with major depressive disorder: a systematic review and meta-analysis
Source: Psychol Med. 2020 Dec 3;51(1):1–10. doi: 10.1017/S0033291720004729 (PMC7856413; doi:10.1017/S0033291720004729)

**Supplementary Figure 1.** Risk of Bias Graph and Summary for Each Included Study


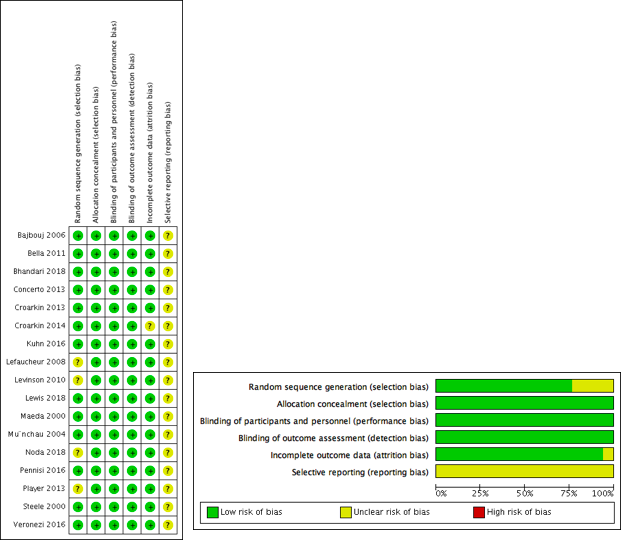


**Supplementary Figure 2.** PRISMA Diagram: flowchart mapping out the inclusion and exclusion process of records.


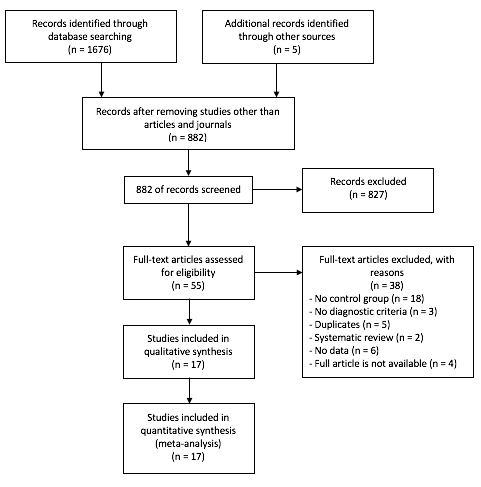


**Supplementary Figure 3.** The Results of Meta-Regression Analyses on Patients’ Age.

**(a) meta-regression of patients’ age and SMDs of SICI**

**(b) meta-regression of patients’ age and SMDs of CSP**

**(c) meta-regression of patients’ age and SMDs of ICF**

**Supplementary Figure 4.** The Results of Meta-Regression Analyses on Patients’ Gender Rate (%Female).

**(a) meta-regression of patients’ gender (% female) and SMDs of SICI**

**(b) meta-regression of patients’ gender (% female) and SMDs of CSP**

**(c) meta-regression of patients’ gender (% female) and SMDs of ICF**

**Supplementary Figure 5.** The Results of Meta-Regression Analyses on HRSD-17 Score.

**(a) meta-regression of HRSD-17 score and SMDs of SICI**

**(b) meta-regression of HRSD-17 score and SMDs of CSP**

**(c) meta-regression of HRSD-17 score and SMDs of ICF**

**Supplementary Figure 6.** Funnel Plots of Studies Included in Each Paradigm.

1. **SICI**


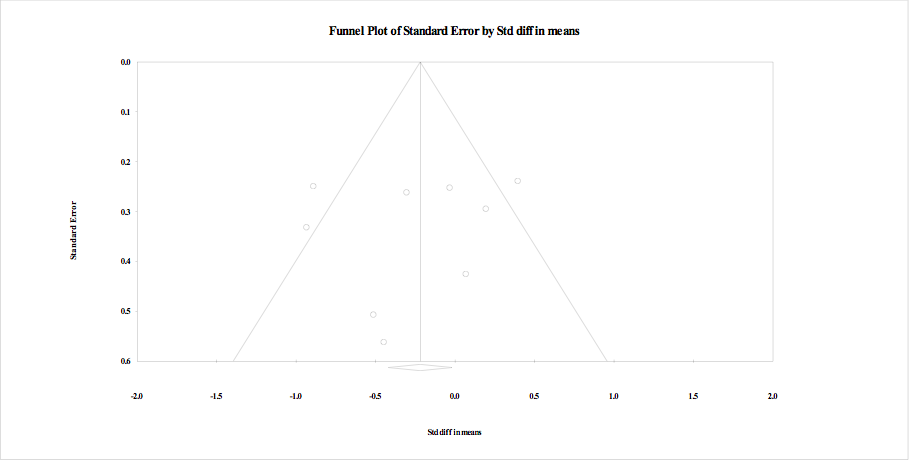


1. **CSP**


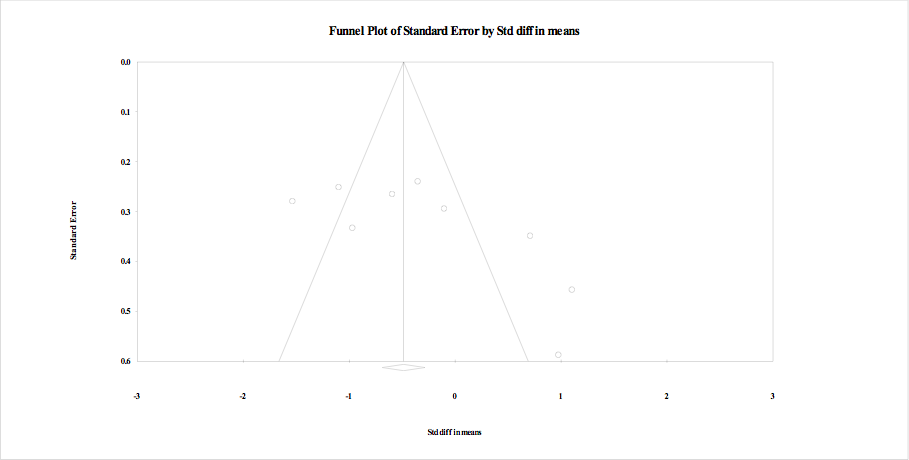


1. **ICF**


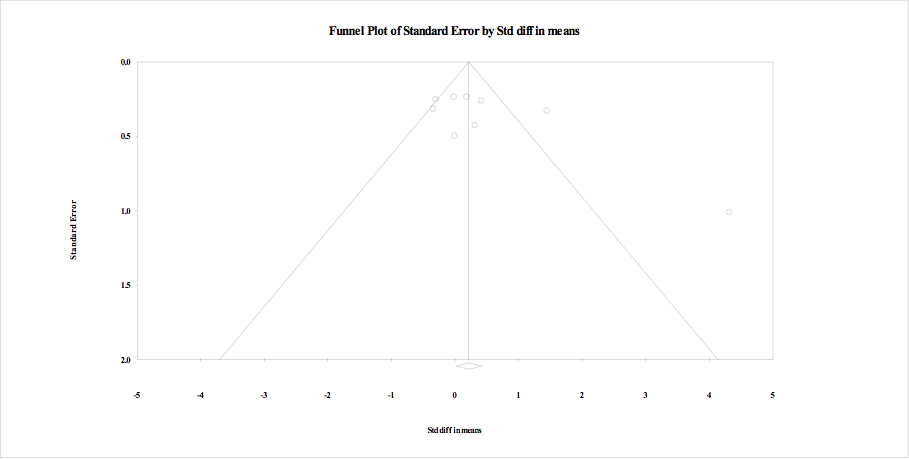


1. **PAS**


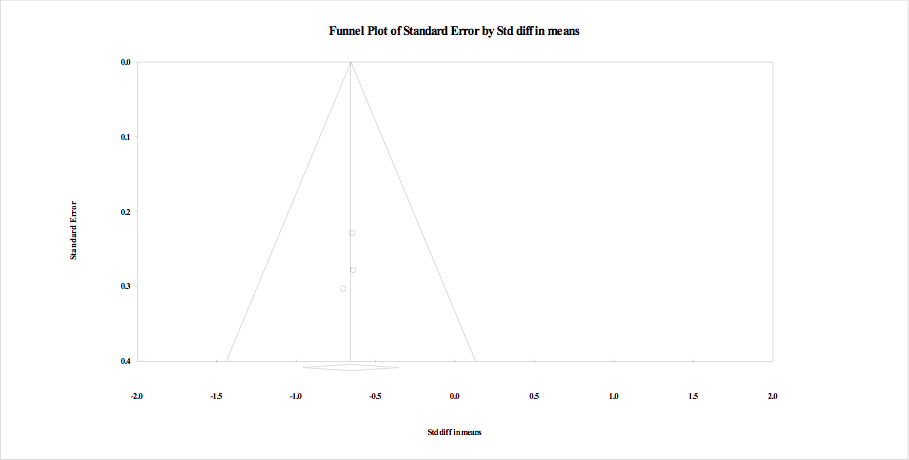

Supplement: Supplementary file 1 [file S0033291720004729sup.zip › S0033291720004729sup002.docx]
