# Supplementary material for: Transcranial magnetic stimulation neurophysiology of patients with major depressive disorder: a systematic review and meta-analysis
Source: Psychol Med. 2020 Dec 3;51(1):1–10. doi: 10.1017/S0033291720004729 (PMC7856413; doi:10.1017/S0033291720004729)
Supplement: Supplementary file 1 [file S0033291720004729sup.zip › S0033291720004729sup001.doc]

**PRISMA Flow Diagram**

**Screening**

**Included**

**Eligibility**

**Identification**

Records identified through database searching
(n = 1676)

Additional records identified through other sources
(n = 5)

Records after removing studies other than articles and journals
(n = 882)

882 of records screened

Records excluded
(n = 827)

Full-text articles assessed for eligibility
(n = 55)

Full-text articles excluded, with reasons
(n = 39)

- No control group (n = 18)

- No diagnostic criteria (n = 3)

- No TMS-EMG study (n = 1)

- Duplicates (n = 5)

- Systematic review (n = 2)

- No data (n = 6)

- Full article is not available (n = 4)

Studies included in qualitative synthesis
(n = 16)

Studies included in quantitative synthesis (meta-analysis)
(n = 16)
